# Influence of Polymer-Clay Interfacial Interactions on the Ignition Time of Polymer/Clay Nanocomposites

**DOI:** 10.3390/ma10080935

**Published:** 2017-08-11

**Authors:** Indraneel S. Zope, Aravind Dasari, Zhong-Zhen Yu

**Affiliations:** 1 School of Materials Science and Engineering, Nanyang Technological University, Singapore 639798, Singapore; zopeis@ntu.edu.sg; 2 State Key Laboratory of Organic-Inorganic Composites, College of Materials Science and Engineering, Beijing University of Chemical Technology, Beijing 100029, China; yuzz@mail.buct.edu.cn

**Keywords:** time-to-ignition, thermo-oxidation, combustion, polyamide 6, clay

## Abstract

Metal ions present on smectite clay (montmorillonite) platelets have preferential reactivity towards peroxy/alkoxy groups during polyamide 6 (PA6) thermal decomposition. This changes the decomposition pathway and negatively affects the ignition response of PA6. To restrict these interfacial interactions, high-temperature-resistant polymers such as polyetherimide (PEI) and polyimide (PI) were used to coat clay layers. PEI was deposited on clay by solution-precipitation, whereas PI was deposited through a solution-imidization-precipitation technique before melt blending with PA6. The absence of polymer-clay interfacial interactions has resulted in a similar time-to-ignition of PA6/PEI-clay (133 s) and PA6/PI-clay (139 s) composites as neat PA6 (140 s). On the contrary, PA6 with conventional ammonium-based surfactant modified clay has showed a huge drop in time-to-ignition (81 s), as expected. The experimental evidences provided herein reveal the role of the catalytic activity of clay during the early stages of polymer decomposition.

## 1. Introduction

Polymer/clay nanocomposites show a significant reduction in peak heat release rates (up to 70%) during combustion [1,2]. However, with the exception of a few reports [3,4], the majority of the studies on flammability of polymer/clay nanocomposites have reported an earlier ignition behavior compared to their corresponding neat polymers [2]. It is known that the reactivity of smectite clays such as montmorillonite is affected by replacing the relatively mobile and exchangeable cationic species, such as Na^+^ or Ca^2+^, on their surfaces by higher valence metal ions, such as Mg^2+^, Zn^2+^, Cu^2+^, Al^3+^ or Fe^3+^ [5,6,7,8]. In fact, this attribute has been exploited for applications like catalysis [9,10]. In our recent systematic investigations on this topic [7,11], we showed that by amplifying the content of a specific cation (which are inherently present in the clay structure), thermal decomposition and combustion reactions of PA6 could be affected. For instance, Al^3+^-rich PA6/clay nanocomposites ignited early with a lower onset temperature of decomposition under oxidative conditions; while an Mg^2+^-rich composite displayed the maximum thermo-oxidation stability and gave the highest residue. This suggested that the chemical interaction of the PA6 matrix and/or its intermediate decomposition products with the clay surface changes the decomposition pathway of PA6. Furthermore, it has been observed that the nature of intermediate products depends on the type of interacting and/or coordinating metal ions.

Investigations have also been carried out on the usage of other metal ions like nickel along with clay layers to influence the carbonaceous residue formation [12,13,14]. Tang et al. [12] have noted that the yield of multiwalled carbon nanotubes increased (up to 41.1% from 5.2%) in the presence of nickel and clay in polypropylene when subjected to catalytic combustion. Hu et al. [14] have also used clay supported with nickel cation to enhance char-forming reactions in polypropylene (PP) containing ammonium polyphosphate (APP), melamine formaldehyde (MF), and 1,3,5-triazine-2,4,6-trimorpholinyl (TTM). Though the exact reasons are unknown, it has been shown that even the addition of 3 wt % of Ni^2+^/MF modified montmorillonite (MMT) to a PP/APP/TTM system results in a drop of ignition time from 38 s to 31 s. In the presence of a non-swelling clay such as sepiolite (0.5 wt %), Pappalardo et al. [15] have noted a reduction in time-to-ignition and limiting oxygen index (LOI) of PP. However, they have attributed this to the reduced melt flow due to the increase in viscosity of PP in the presence of sepiolite.

Based on the above discussions, it is hypothesized that if there is a delay in the direct chemical interactions between the clay surface and the neighboring decomposing organic matrix, the effect on time-to-ignition could be minimized. This forms the basis of the current work, which attempts to validate the hypothesis. A simple approach is to coat the clay layers with another matrix before blending with PA6. In fact, though not directly related to catalytic activity or reactivity, there are some reports on the coating of clay platelets (which are inherently highly hydrophilic) with various water-soluble and soft polymers such as polyvinyl alcohol [16], polypyrole [17], and polyethyleneglycol [18]. For instance, coated and self-assembled clay sheets were synthesized to mimic nacre-like building blocks with intrinsic hard/soft character [16]. Nonetheless, the poor thermal stability and water solubility of these polymers hinder their usage to prove the above-proposed hypothesis. It is essential to use high-temperature-resistant polymers that are compatible with PA6 and maintain the protective coating on clay during compounding as well as the initial stages of thermal decomposition of the PA6 matrix (i.e., polymers with a glass transition temperature above 220 °C). Accordingly, polar polymers such as PEI and PI were chosen. The rationale behind using these polymers is to compare the effectiveness of two coating methodologies, i.e., solution blending-precipitation (for PEI) and solution blending-imidization-precipitation (for PI). Commercially available organically modified clay, especially ammonium-based surfactant modified clay (C30B), has been extensively studied in the PA6 matrix for combustion properties. Hence, the use of C30B instead of unmodified/pristine Na^+^MMT provides an opportunity to have a direct comparison with ignition times previously reported for PA6/organoclay systems. This will facilitate a straight-forward evaluation of the proposed hypothesis.

## 2. Results and Discussion

### 2.1. Analysis of Clay Coatings

**Figure 1:**
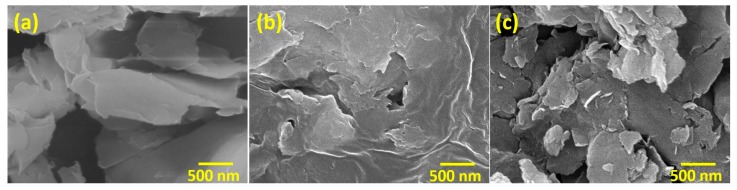
Representative scanning electron microscope micrographs: (**a**) organoclay; (**b**) PEI-clay; and (**c**) PI-clay.

Representative scanning electron microscope (SEM) micrographs of organoclay (i.e., C30B), PEI coated clay (PEI-clay) and PI coated clay (PI-clay) platelets are shown in Figure 1, highlighting the differences in the uniformity of coating on organoclay with PEI and PI. The precipitation of PEI from solution on organoclay resulted in a non-uniform and thick coverage, whereas imidization of poly(pyromellitic dianhydride-co-4,4′-oxydianiline) amic acid (PAA) over organoclay resulted in a more uniform coverage. This is due to the good dispersion and distribution of the silicate layers in PAA, providing a high heterogeneous surface area for further reactions. In fact, this uniformity in the coating with PI is expected, based on the investigation by Tyan et al. [19]. They reported that the imidization kinetics of PAA are enhanced with the addition of organically (p-phenylenediamine) modified clay. This further reduced the imidization temperature from 300 °C to 250 °C without affecting the structure of the PI thus formed. The heterogeneous surface provided by the clay platelets was considered as a major parameter for changes in reaction temperature and kinetics, as it promoted dehydration and imide ring closure reactions.

**Figure 2:**
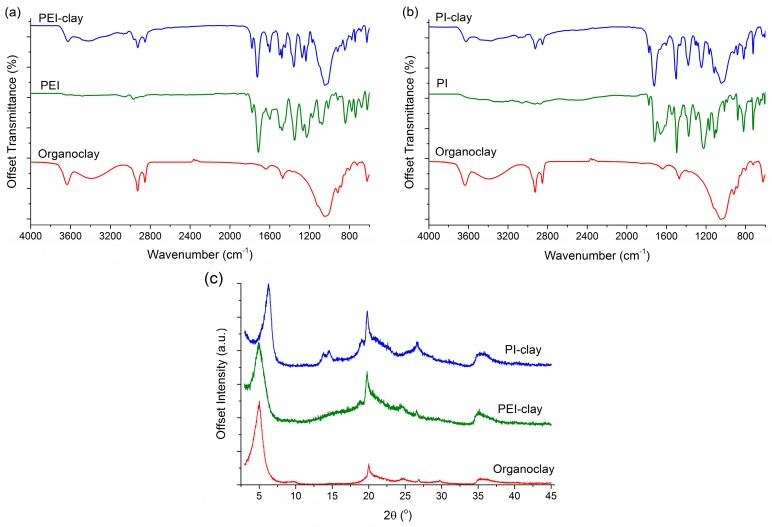
FTIR spectra of (**a**) organoclay, PEI, and PEI-coated clay; (**b**) organoclay, PI, and PI-coated clay; and (**c**) XRD patterns of organoclay, PEI- and PI-coated clay powders.

Further, the FTIR spectra of coated clay powders in Figure 2a,b confirm the completion of reactions. Characteristic IR bands for imide I (anti-symmetric C=O stretch) and imide II (symmetric C=O stretch) are seen at 1800 cm^−1^ and 1730 cm^−1^, respectively. Imide III (C–N stretch) is also present at 1385 cm^−1^ in coated clays, which confirms the deposition of PEI and PI on clay. Detailed peak assignments are listed in Table 1. XRD patterns of the coated clay powders indicate intercalated structures in both cases (Figure 2c). This is expected based on the above discussions and SEM micrographs, confirming the rather thick coverage of polymer on clay stacks. Observed d_001_ spacing for organoclay, PEI-clay, and PI-clay are 17.6 Å, 18 Å, and 14.2 Å, respectively. It should be noted that equivalent amounts of clay and (coating) polymer (i.e., 1:1 wt % ratio) are employed here, which is possibly one of the reasons for this behavior.

### 2.2. Thermal Decomposition Behavior of Coated Clays and Their PA6 Nanocomposites

**Figure 3:**
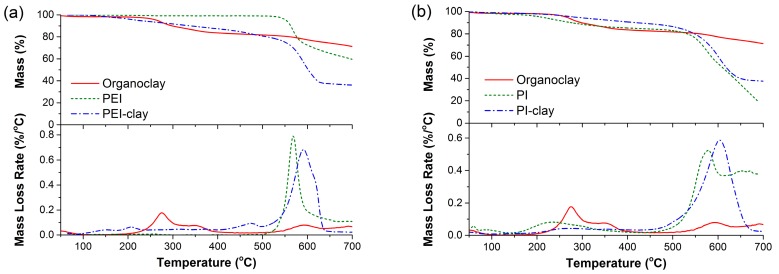
Thermogravimetric curves under oxidative conditions: (**a**) organoclay, PEI, and PEI-coated clay; and (**b**) organoclay, PI, and PI-coated clay.

Thermal stability of the different clay powders was analyzed using TGA (thermogravimetric analysis) under oxidative conditions (Figure 3). With organoclay, as reported in many studies, major mass loss is seen between 200–350 °C, which is attributed to the loss of excess and surface adsorbed surfactant molecules. Upon continued heating, the intercalated surfactant degrades (under confined conditions of collapsed platelets) to form carbonaceous residue. This residue oxidizes completely above 550 °C. In the case of neat PEI, no mass loss is seen up to 535 °C, whereas (synthesized) PI displays a mass loss of around 14% between 180–300 °C. This is primarily due to the imidization of residual PAA with the evolution of water (this is validated by the presence of the IR band for PI at 1650 cm^−1^ in Figure 2b, which is associated with carbonyl stretch from amic acid).

**Figure 4:**
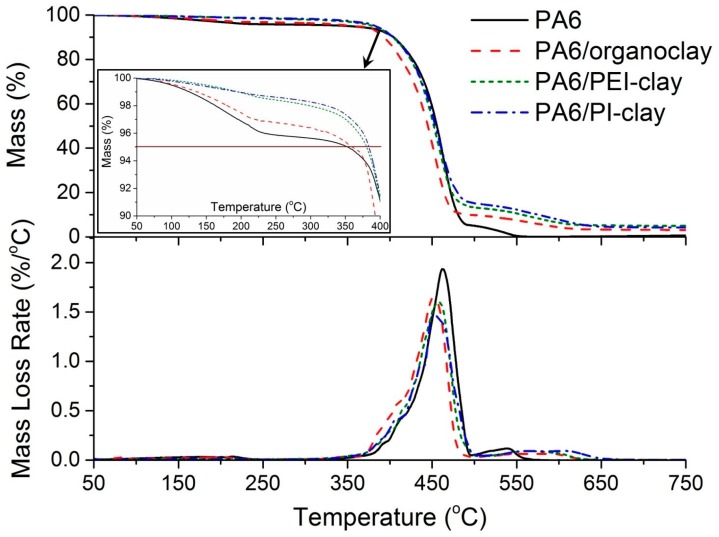
Thermogravimetric curves under oxidative conditions for neat PA6 and its clay composites.

With both PEI- and PI-coated silicate platelets/stacks, a gradual mass loss is seen starting from 100 °C until 450 °C. The mass loss associated with this step is around 16% for PEI-coated clay and 11% for PI-coated clay. This behavior can be attributed to the continuous loss of fragmented surfactant molecules as a consequence of solution treatment experienced by organoclay. Both PEI- and PI-coated clays display an overlap of major mass loss peaks with their respective coating polymers beyond 530 °C. The key point to note here is the good thermal stability of PEI- and PI-coated clay platelets, which effectively retards the direct chemical interactions between the clay surface and the neighboring decomposing PA6 matrix. This is evident from the higher onset decomposition temperatures (T_5%_) for PA6/PEI-clay and PA6/PI-clay composites, as compared to those of the PA6/organoclay system (Figure 4 (inset) and Table 2). However, there is no significant difference in their T_50%_ values, suggesting a similar rate of mass loss post-decomposition onset.

### 2.3. Combustion Response of PA6 with Coated Clays

**Figure 5:**
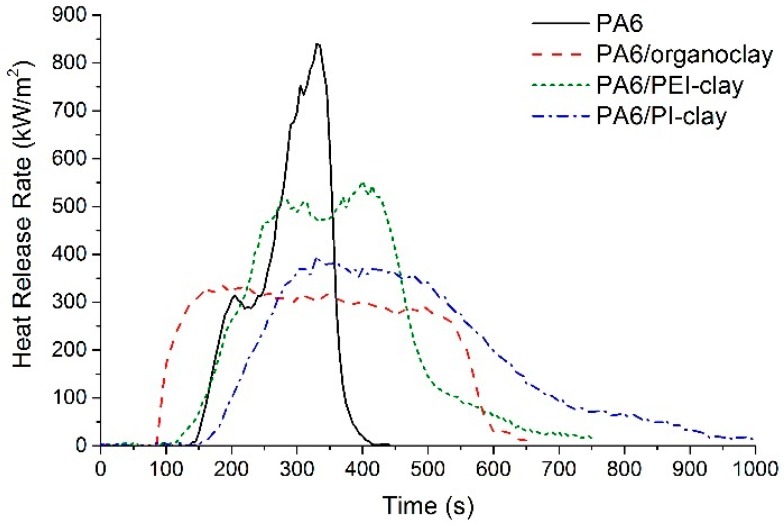
Heat release rate (HRR) curves for PA6 and its composites, as obtained from a cone calorimeter when exposed to a heat flux of 35 kW/m^2^.

PA6/organoclay nanocomposite shows early ignition by almost 59 s as compared to neat PA6 (Figure 5 and Table 3). Coated clay composites display significant improvement in ignition resistance, and their ignition times are similar to neat PA6. This confirms that the clay-matrix interaction (as proposed in our previous investigation [11]), a combined effect of Brønsted and Lewis acid characteristics associated with the metal ions, plays a major role in the ignition behavior of polymer/clay nanocomposites. That is, during the pre-ignition stage of PA6 in particular, the chemical inertness of PEI and PI prevent clay from interacting with the decomposing PA6. This allows the matrix to continue to follow the peroxy-based decomposition pathway under thermo-oxidative conditions, similar to neat PA6. As a result, the time-to-ignition of PEI- and PI-coated clay composites is similar to that of neat PA6. In contrast, we have previously shown that when the clay is exposed to the decomposing PA6 matrix, metal ions (from the clay) have preferential reactivity towards peroxy/alkoxy groups. Metal ions can either oxidize by reducing the hydroperoxide group into a more reactive peroxy group, or reduce by oxidizing the hydroperoxide group into another reactive alkoxy group. This ultimately could result in alternate pathways for the decomposition of PA6.

**Figure 6:**
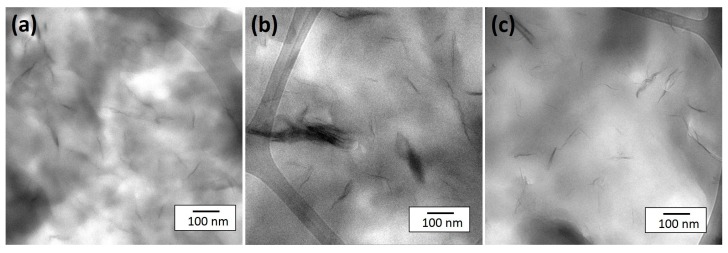
Representative transmission electron microscope images of: (**a**) PA6/organoclay; (**b**) PA6/PEI-clay; and (**c**) PA6/PI-clay.

Apart from the significant positive effect on the time-to-ignition, other advantages of coated clay systems include the lower THR and smoke generation in comparison with PA6/organoclay composite. The PA6/PEI-clay composite also shows a 34% reduction in peak HRR value compared to neat PA6, whereas the PA6/PI-clay composite shows a 52% reduction, very similar to the 60% reduction for the PA6/organoclay nanocomposite. The variations in pHRR follow a pattern that can be correlated back to the coating uniformity, dispersion, and distribution of coated clay stacks/platelets in the PA6 matrix. To support this argument, TEM micrographs showing the extent of the dispersion of clay platelets are shown in Figure 6. The micrographs clearly reveal that in the PA6/PEI-clay composite, many clusters/stacks of clay platelets are seen in addition to some finely dispersed clay. As discussed in many studies before [22,23,24], dispersion and distribution of high aspect ratio nanoparticles like clay are key parameters for obtaining significant reductions in the peak HRR. This is due to the dependence on the physical barrier formed by collapsing clay platelets during combustion. However, it is important to note that the dispersion/distribution of clay in PA6 is also influenced by the affinity of PEI and PI to PA6. On a similar note, Morgan et al. [25] synthesized PEI/clay nanocomposites via in situ imidization of PAA (for PEI) and with organically modified clay (with an aliphatic chain of twelve carbons). Even with 10 wt % clay loading (as opposed to 50 wt % clay loading in the current system), the magnitude of intercalation observed was poor. It was concluded that in situ imidization produced a mixed dispersion state in the PEI matrix.

Smoke generation is another critical parameter during combustion, especially during the early stages that are key for safe evacuation. When compared with PA6, PA6 composites with PEI-clay and PI-clay produce significantly less smoke during the pre-ignition stages, as seen from Table 3. The PA6/organoclay composite rapidly generates smoke even prior to ignition. This further corroborates the effectiveness of coated clays.

## 3. Materials and Methods

### 3.1. Materials

Ultramid B3S (trade name PA6) with a melting point of 220 °C and a V-2 rating in UL94 (vertical burning test) at a thickness of 1.6 mm was supplied by BASF, Singapore. Prior to melt compounding, PA6 was dried at 90 °C for 24 h in a convection oven. Montmorillonite (clay), organically modified with methyl, tallow, bis-2-hydroxyethyl quaternary ammonium salt, commercially known as Cloisite^®^ 30B or C30B, was procured from BYK-Chemie GmbH (Singapore). Commercially available solvent-soluble ULTEM 1000 PEI was obtained from SABIC Innovative Plastics (Singapore). It has a reported T_g_ of ~217 °C and a melting range of 320–355 °C. However, for the PI coating, as it is known for its insolubility, poly(pyromellitic dianhydride-co-4,4′-oxydianiline)amic acid (PAA) solution, a precursor for PI, was obtained from Sigma-Aldrich (Singapore) at a concentration of 15 ± 5 wt % in N-methyl-2-pyrrolidone/aromatic hydrocarbons (80%/20% solvent ratio). This was followed by imidization, resulting in PI. PI, thus formed, had a T_g_ > 300 °C. It does not melt or decompose at temperatures below 540 °C. Other solvents required for coating such as tetrahydrofuran (THF) and N, N-dimethylacetamide (DMAc) were procured from Merck Millipore (Singapore).

### 3.2. Synthesis of Coated Clays: PEI-Clay and PI-Clay

**Figure 7:**
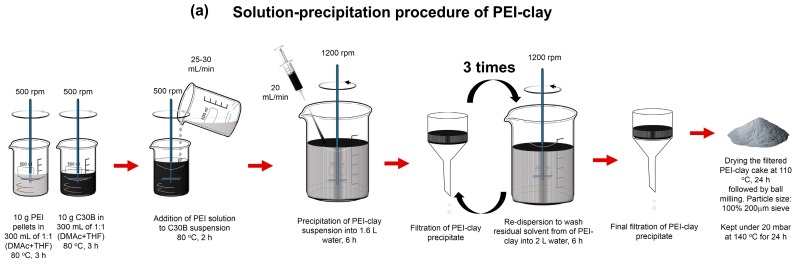

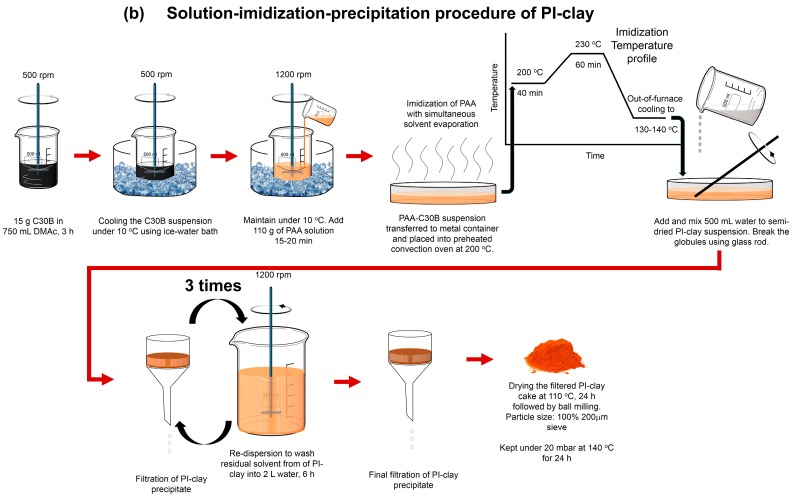
Step-by-step synthesis procedure: (**a**) PEI-clay platelets by solution-precipitation technique; and (**b**) PI-clay platelets by solution-imidization-precipitation technique.

A schematic flowsheet of detailed step-by-step procedure that was followed to obtain PEI- and PI-coated clay powders is given in Figure 7. In general, a suspension of C30B in organic solvent was mixed with dilute solution of PEI (or PAA) to achieve 1:1 wt. ratio, followed by precipitation in non-solvent (deionized water). An additional step of imidization was carried out prior to filtration in the case of the PI-coating. PEI-clay and PI-clay, thus obtained, were used for further characterization. To achieve the same degree of physical barrier effect across all composites (as influenced by the loading level of clay platelets), the loading level of base clay was kept constant. Accordingly, 5 wt % of organoclay and 10 wt % of coated clays were used in the fabrication of PA6/clay nanocomposites.

### 3.3. Extrusion Parameters

Melt mixing of coated clays with PA6 was carried out using the Leistritz Micro 18 twin screw extruder (Singapore) at 120 rpm to assist the uniform dispersion of clay platelets/stacks. The extrusion temperature profile used was: (hopper end) 230–235–240–245–245–250 °C (die end). Pelletized material was compression molded into 100 mm × 100 mm × 5 mm sized samples using a Carver Hot Press (Wabash, IN, USA.) maintained at 250 °C with a molding pressure of 5 bar and a total molding time of 20 min.

### 3.4. Characterization

X-ray diffraction studies of clay powders and molded polymer composites were carried out on a Shimadzhu XRD 6000 (Singapore, 40 kV, 30 mA, Cu kα, λ = 1.542 Å) with a scan speed of 2°/min, scan range of 3–45°, and step size of 0.02°. Fourier transform infrared spectra of PA6 materials, clay, and powdered residues (prepared using KBr pellets) were collected using a Perkin-Elmer SpectrumGX spectrometer (Singapore) and Perkin-Elmer Frontier Spectrometer (Singapore, surface Attenuated Total Reflectance (ATR) FTIR). All spectra were acquired in the range of 400–4000 cm^−1^ using 32 scans and resolution of 4 cm^−1^. To observe clay dispersion in the polymer matrix, trimmed polymer composite samples were ultra-microtomed at 0.2 mm/s using a diamond knife on Leica Ultracut UCT (Singapore) in liquid N_2_ environment at –100 °C to obtain 70–90-nm thick sections. The sections were picked up using a droplet of 2.3 mol sucrose and placed on formvar/carbon-coated 400-mesh copper grids. After thorough rinsing with distilled water to wash away the sucrose, sections were observed using a Carl Zeiss transmission electron microscope (Singapore), LIBRA 120, in bright field mode, at an accelerating voltage of 120 kV. The morphology of uncoated and coated clays was observed using a JEOL field emission scanning electron microscope, FESEM 6340F (Singapore) with an accelerating voltage of 10–20 kV and a working distance between 7–9 mm. All samples were sputter-coated with platinum for 10 s, at a distance of 30 mm from the source and with a voltage of 20 kV.

### 3.5. Thermo-Oxidation and Combustion Tests

Thermogravimetric analysis (TGA) for different clay powders and polymer composites was carried out on TA Instruments Q500 (Singapore) under air atmosphere. Clay samples were pre-dried at 110 °C, while polymer samples were dried at 80 °C for 24 h in a convection oven. Tests were carried out from room temperature to 750 °C employing a heating rate of 20 °C/min. A cone calorimeter from Fire Testing Technology (East Grinstead, West Sussex, UK) was used to determine the combustion response of compression molded PA6 and its composites. The tests were carried out according to ISO 5660 at an irradiant heat flux of 35 kW/m^2^.

## 4. Conclusions

To retard the catalytic effect of clay on the time-to-ignition of a PA6 matrix, a methodology that prevented direct chemical interactions between the clay surface and the neighboring decomposing PA6 matrix was employed here. This involved the coating of clay with thermally stable polymers such as PEI and PI by solution blending-precipitation and solution blending-imidization-precipitation techniques, respectively. The thermal decomposition and combustion behavior of the composites confirmed the delayed decomposition onset as well as the time-to-ignition for coated clay composites. Additionally, up to 52% reduction in peak HRR was noted for coated clay composites relative to neat PA6, maintaining the advantage of the presence of clay platelets. This work proved that preventing direct contact between the clay surface and the decomposing PA6 matrix using a physical barrier such as a high-temperature-resistant polymer coating is a simple solution to the major issue of early ignition with polymer/clay nanocomposites.

## Figures and Tables

**Table 1:** FTIR bands assignment for organoclay and coating polymers (PI, PEI) [20,21].

Material	Absorption Range (cm^−1^)	Assignment
Organoclay	3650	Al–Al–OH in MMT, stretch
Organoclay	3250–3500	–OH in MMT, stretch
Organoclay	2950–2830	–CH_3_, –CH_2_– aliphatic carbon, stretch
PI, PEI	1800	Imide I C=O, anti-sym. stretch
PI, PEI	1740–1720	Imide II C=O, sym. stretch
PI, PEI	1610–1600	C=C in aromatic, stretch
PI, PEI	1500–1490	aromatic ring stretch
PI, PEI	1390–1380	Imide III C–N, stretch
PEI	1250	Tertiary C aliphatic
PI, PEI	1230–1210	C–O–C aromatic ether
Organoclay	1150–1050	Si–O–Si in MMT, stretch
PI, PEI	~730	Imide IV C–N ring deformation

**Table 2:** T_5%_, T_50%_, and T_P_ from thermogravimetric analysis thermograms for PA6 and its composites.

Specimen	T_5%_ (°C)	T_50%_ (°C)	T_P_ (°C)
PA6	353	455	462
PA6/organoclay	360	445	451
PA6/PEI-clay	380	452	458
PA6/PI-clay	384	454	453

**Table 3:** Cone calorimeter results of neat PA6 and its composites.

Specimen	TTI (s)	pHRR (kW/m^2^)	THR (MJ/m^2^)	Char (%)	TSP at Ignition (m^2^)
PA6	140	840	97	0	0.58
PA6/organoclay	81	338	142	13.1	0.70
PA6/PEI-clay	133	554	122	7.6	0.13
PA6/PI-clay	139	396	128	9.4	0.47

TTI: time-to-ignition; pHRR: peak values of HRR; THR: total heat released; TSP: total smoke produced.
